# Particular hyperpigmentation of the soft palate

**DOI:** 10.11604/pamj.2018.29.187.15110

**Published:** 2018-04-02

**Authors:** Cinzia Casu, Luca Viganò

**Affiliations:** 1 Private Dental Practice, Cagliari, Italy; 2 University of Milan, Department of Radiology, Milano, Italy

**Keywords:** Oral pigmentation, palate lesion, smoke pigmentation

## Image in medicine

A 64-year-old patient went to our private practice for a follow-up visit. The medical history reported mild hypertension, she was taking statins to control cholesterol and she reported to be positive for hepatitis B without however having had symptoms and signs of disease. She accidentally discovered through blood tests performed many years ago, to have contracted the HBV infection without realizing it and having overcome it. She has undergone tonsillectomy in adolescence. She had a very poor oral hygiene condition, partial upper and lower removable prostheses, and is a strong smoker (more than 20 cigarettes a day for decades). The patient presented dark pigmentations on the bilateral cheeks. On the soft palate there were pigmented areas, very large, brown in color and the patient, said to have noticed them since young, and that have not undergone evolution. The diagnostic hypothesis was smoke hyperpigmentation and probably the presence of the prosthesis did not allow the manifestation of it in the gingival tissues and in the hard palate. Pigmentations on the palate could be diagnosed with oral nevi, drug pigmentations or oral melanoma. We decided for a closely follow up of the patient, who did not want to be biopsied. We informed her about the possible risks of developing squamous cell carcinoma in heavy smokers.

**Figure 1:**
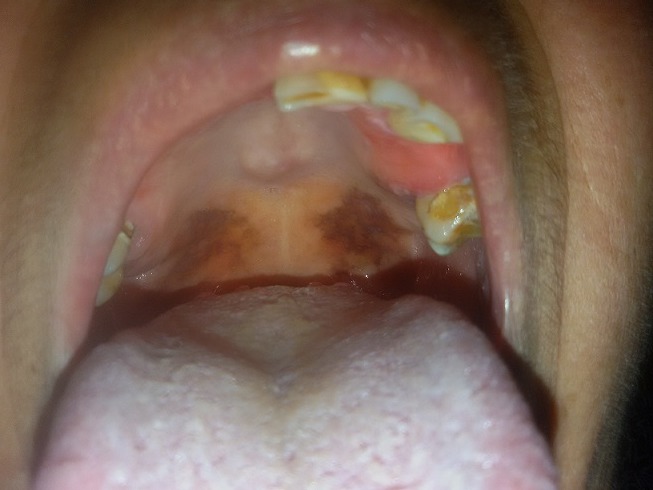
hyperpigmentation of the soft palate

